# Single-Cell Transcriptome Profiling Reveals Neutrophil Heterogeneity and Functional Multiplicity in the Early Stage of Severe Burn Patients

**DOI:** 10.3389/fimmu.2021.792122

**Published:** 2022-01-18

**Authors:** Jiamin Huang, Zhechen Zhu, Dongdong Ji, Ran Sun, Yunxi Yang, Lu Liu, Yiming Shao, Yi Chen, Linbin Li, Binwei Sun

**Affiliations:** ^1^ Department of Burns and Plastic Surgery, Affiliated Suzhou Hospital of Nanjing Medical University, Suzhou, China; ^2^ School of Medicine, Jiangsu University, Zhenjiang, China

**Keywords:** neutrophil, heterogeneity, function, single-cell transcriptome profiling, burn

## Abstract

The pathophysiological mechanisms, especially the roles of immune cells, underlying early stages of severe burn injury have not yet been fully clarified. Here, we analyzed circulating neutrophils (PMNs) in healthy donors and early burned patients by single-cell RNA sequencing to provide a comprehensive transcriptional landscape of PMNs in heterogeneity and functional multiplicity. Circulating PMNs in the healthy donors and burned groups were divided into five subgroups (G3, G4, G5a, G5b, G5c) with different functions. The dominant subsets of PMNs in homeostasis and burn injury significantly differed between groups. In addition, cells in the same subpopulation had the same core identity markers but performed different functions in healthy and burned states. Under burned conditions, PMN activation was very evident and accompanied by clear degranulation and metabolic abnormalities. Interestingly, was found that PMN activation, degranulation, chemotaxis, phagocytosis and reactive oxygen species (ROS) production in burned patients significantly differed between day 1 and days 2 or 3, thus providing a theoretical basis for PMN interventions in early burn stages. Significantly, previously undescribed transcription factors were also identified, including ZNF-787, ZNF-467, ZNF-189, ZNF-770, ZNF-262. In conclusion, this study conducted for the first time a detailed analysis of the heterogeneity and functional multiplicity of PMNs in early stages of severe burn injuries. Our findings attempted to clarify the influence of PMN heterogeneity on the pathophysiology and related mechanisms of burn injuries, which can provide new ideas for further research in burn intervention.

## Introduction

Burn injury is a common form of trauma (1). Burn wound prognosis depends on the severity of the burn injuries. Mild burn injuries often heal on their own, but severe or very severe burn injuries can endanger the lives of patients (2). Severe burn injuries refer to a total area greater than 30% or a third-degree burn area greater than 10% (3). Patients with extensive burn injuries are often in shock when they are admitted to the hospital, because of impaired skin protection, which induces systemic changes such as tissue fluid outflow, blood volume reduction and tissue damage. Active fluid rehydration is a common clinical method used to address shock, which can restore blood perfusion and decrease ischemic damage to cells, tissues and organs (4). However, burn injuries, particularly large area burn injuries, represent a complex process. Beyond massive fluid loss, the body is under a pathological condition involving excessive inflammation, hypermetabolism and an acute immune response (5, 6). Early treatment is key to improving prognosis and can decrease systemic inflammatory response syndrome, sepsis, multiple organ failure and other life-threatening conditions in patients (7). Although simple fluid rehydration can relieve the shock state of the body, it does not appear to fundamentally alleviate the disorder within the internal environment of the body, particularly the immune microenvironment (8), which also creates favorable conditions for uncontrolled infection.

As the first line of defense, PMNs exert phagocytosis, degranulation and the release of ROS or neutrophil extracellular traps (9–12). Within hours after burn injury, excessive PMNs are mobilized from the bone marrow into the peripheral blood. However, according to broad consensus, in early burn stages, the body is in the shock phase and has not entered the subsequent infection phase (13). Few researchers have observed the role of excessive PMNs in peripheral blood.

Single-cell RNA sequencing (scRNA-seq) is a powerful tool for characterizing subsets of immune cells and their corresponding functions (14, 15). To explore the heterogeneity and functional changes in PMNs in severely burned patients, we used single-cell sequencing to detect circulating PMNs in healthy and burned groups. In addition, to explore the dynamic changes in neutrophil function in early burn stages, we analyzed patient PMNs in the first 3 days after burn injuries.

## Results

### Classification of PMNs in Early Burn Injuries

We reviewed the electronic medical records of patients with extensive burn injuries (total body surface area > 30%, degree II–III) between January 2010 and December 2020. The proportion of PMNs in severely burned patients was nearly 85%, whereas that in healthy people was approximately 40%–75% (**Table 1**). To investigate the functional changes in PMNs in early stages of severe burn injuries, we performed scRNA-seq of circulating PMNs in the healthy and burned groups. Sequencing samples were collected from the peripheral blood of five healthy controls and five patients (1, 2 and 3 days) after severe burn injuries (**Table 2**). To obtain a complete population of PMNs, we used magnetic bead separation instead of the Ficoll method.

**Table 1:** Characteristics of severe burned patients over the last 10 years.

Characteristics	Burn-Day1	Burn-Day2	Burn-Day3	Reference Ranges
N	n=233	n=233	n=233	N/A
Age	45 (18 ~ 92)	45 (18 ~ 92)	45 (18 ~ 92)	N/A
%TBSA	>30	>30	>30	N/A
Degree of burn	II~III	II~III	II~III	N/A
Sex, male (%)	151 (64.81)	151 (64.81)	151 (64.81)	N/A
WBC count (10^9^/L)	21.21 ± 8.65	18.02 ± 7.61	13.89 ± 5.88	3.50 ~ 9.50
Neutrophil (10^9^/L)	17.81 ± 8.17	15.64 ± 6.99	11.70 ± 5.35	1.80 ~ 6.30
Monocyte (10^9^/L)	1.19 ± 0.65	1.23 ± 0.73	1.09 ± 0.58	3.00 ~ 10.00
Lymphocyte (10^9^/L)	2.07 ± 1.79	1.12 ± 0.67	1.06 ± 0.60	1.10~3.20
Eosinophil (10^9^/L)	0.08 ± 0.14	0.01 ± 0.03	0.01 ± 0.02	0.02~0.52
Basophil (10^9^/L)	0.05 ± 0.04	0.02 ± 0.03	0.02 ± 0.02	0.00~0.06
Neutrophil/WBC(%)	83.51 ± 10.49	85.96 ± 6.12	83.24 ± 7.21	40.00 ~ 75.00

Data presented as median (age range); mean (standard deviation) or number (percentage). N, number; TBSA, Total Body Surface Area; WBC, white blood cell; NA, not applicable.

**Table 2:** Characteristics of healthy controls and patients with burn injuries.

Characteristics	Burn-Day1	Burn-Day2	Burn-Day3	Healthy Control	Reference ranges
N	n=5	n=5	n=5	n=5	N/A
Age	29-61	29-61	29-61	25-32	N/A
%TBSA	>30	>30	>30	N/A	N/A
Degree of burn	II~III	II~III	II~III	N/A	N/A
Sex, male (%)	3 (60)	3 (60)	3 (60)	4 (80)	N/A
WBC count (10^9^/L)	17.94 ± 5.59	12.84 ± 5.37	8.23 ± 4.24	N/A	3.50 ~ 9.50
Neutrophil (10^9^/L)	15.83 ± 5.70	10.26 ± 4.17	6.92 ± 3.86	N/A	1.80 ~ 6.30
Monocyte (10^9^/L)	0.93 ± 0.30	0.85 ± 0.34	0.66 ± 0.34	N/A	3.00 ~ 10.00
Lymphocyte (10^9^/L)	1.14 ± 0.69	0.66 ± 0.05	0.63 ± 0.11	N/A	1.10~3.20
Eosnophil (10^9^/L)	0.02 ± 0.02	0.06 ± 0.12	0.01 ± 0.01	N/A	0.02~0.52
Basophilic (10^9^/L)	0.02 ± 0.01	0.01 ± 0.00	0.01 ± 0.00	N/A	0.00~0.06
Neutrophil/WBC(%)	86.98 ± 5.80	85.58 ± 3.92	82.60 ± 4.86	N/A	40.00 ~ 75.00

Data presented as median (age range); mean (standard deviation) or number (percentage). N, number; TBSA, Total Body Surface Area; WBC, white blood cell; NA, not applicable.

**Figure 1:**
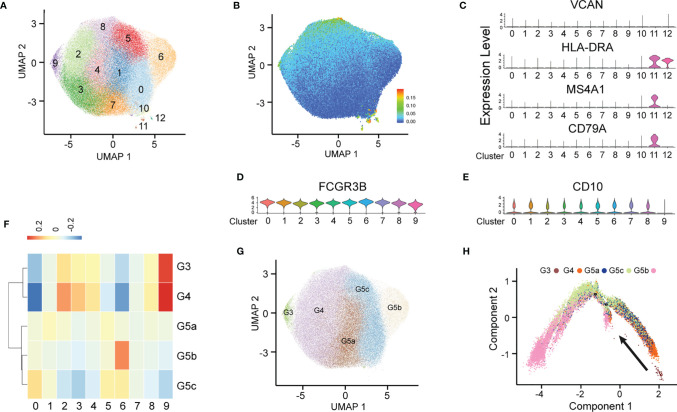
Heterogeneity of peripheral blood PMNs in healthy and severely burned patients. **(A)** UMAP visualization of PMNs. **(B)** The expression of each neutrophil mitochondrial UMI projection on UMAP. **(C–E)** Violin plot of the genes VCAN, HLA-DRA, MS4A1, CD79A, FCGR3B and CD10 in 13 subgroups. **(F)** Correlation of scRNA-seq defined neutrophil populations with the neutrophil subtypes reported by Xie et al. **(G)** Projection of new clustering results on UMAP. **(H)** Results of pseudo-time analysis. Each point represents a PMN. Cellular order was inferred from the expression of the most variable genes in all cells. Trajectory direction was determined by biological development characteristics.

After stringent data quality control (**Supplementary Figure 1A**), we obtained 50000 high-quality cells. Through unsupervised clustering, 13 PMN clusters were distinguished and visualized with uniform manifold approximation and projection (UMAP) (**Figure 1A**). On the basis of known genetic markers (VCAN, HLA-DRA, MS4A1 and CD79A), clusters 10, 11 and 12 were considered B lymphocytes and monocytes (**Figure 1C**). In addition, these three clusters had a high percentage of mitochondrial unique molecular identifier (UMI) count and low UMI count per cell (**Figure 1B** and **Supplementary Figure 1B**). For this reason, clusters 10, 11 and 12 were discarded from further analyses. FCGR3B (CD66b) is a specific surface marker of PMN, and CD10 is a marker of mature PMNs (**Figures 1D, E**). Therefore, subgroups 0–9 were PMNs, and cluster 9 was immature PMNs. Previous studies have divided PMNs in bone marrow, tissue and the circulation into eight cell subsets (G0–G5c). KIT and CD34 are specific markers of granulocyte-monocyte progenitors (16, 17), which are not expressed on the surfaces of PMNs, thus suggesting the absence of a G0–G2 subgroup (**Supplementary Figure 1C**) in peripheral blood PMNs. To dissect neutrophil heterogeneity, we also linked the neutrophil population defined by our scRNA-seq to the data reported by Xie et al. (18) (**Figure 1F**). We also used the ScGeneModule analysis method (**Supplementary Figure 1D**) to re-divide the original ten cell subgroups into G3–G5c, for a total of five PMN subgroups (**Figure 1G** and **Supplementary Figures 1E, F**). Pseudo-time analysis of PMNs was performed with the monocle2 method. As expected, the time trajectory route started from cluster G3 and ended with cluster G5 (**Figure 1H**).

### Relative Conservation of the PMN Population

**Figure 2:**
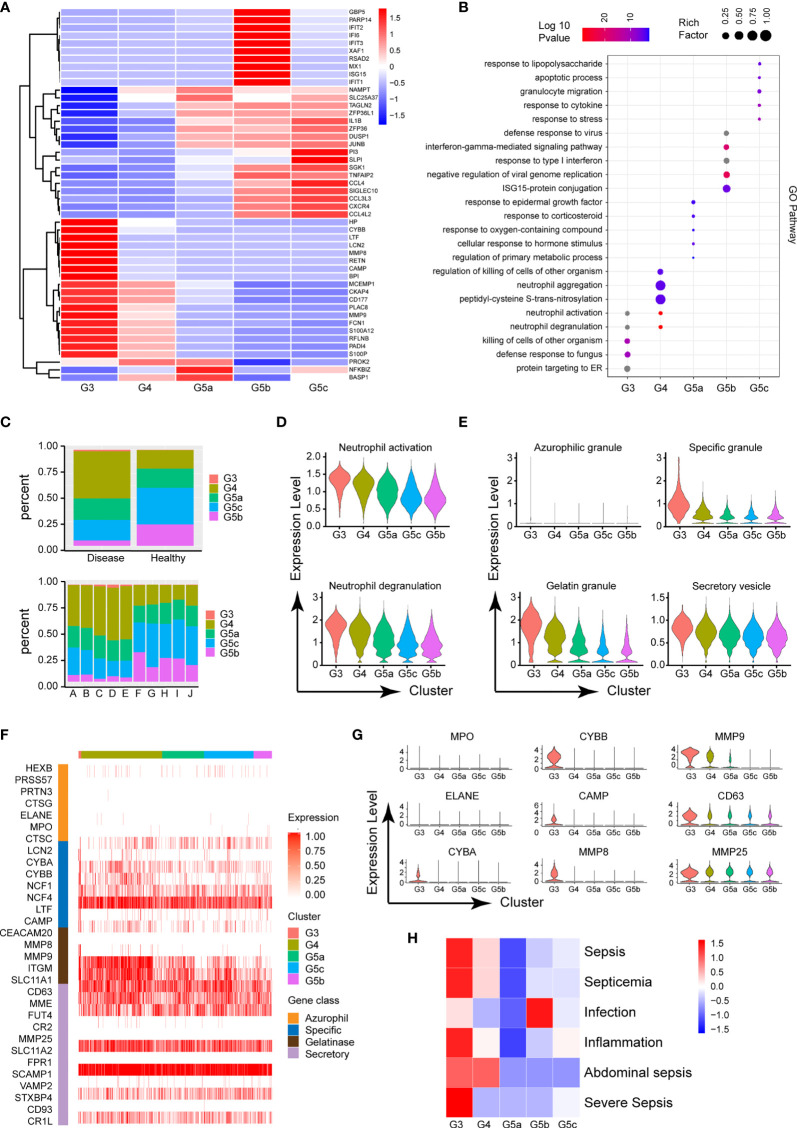
Functional characteristics of each subgroup of PMNs. **(A)** Heatmap of different subsets of PMNs. Red represents high gene expression, and blue represents low gene expression. **(B)** GO (BP) analysis diagram of different genes in each subgroup. The size of the circle represents gene enrichment, and redder color indicates greater functional significance. **(C)** The proportions of five subgroups in different samples. The proportions in the healthy and burned groups (above), and the proportions in each healthy control and burned patient (below). **(D)** Violin plot of the functional scores of activation (above) and degranulation (bottom) of five subpopulations of PMNs. **(E)** The functional scores of four-level particles in each subgroup of PMNs. **(F)** Heatmap showing the expression of neutrophil granule-related genes for all PMNs. **(G)** Violin plot of particle-related gene expression. **(H)** Heatmap of the correlation between each subgroup and the disease. Red represents positive correlation, and blue represents negative correlation.

**Figure 3:**
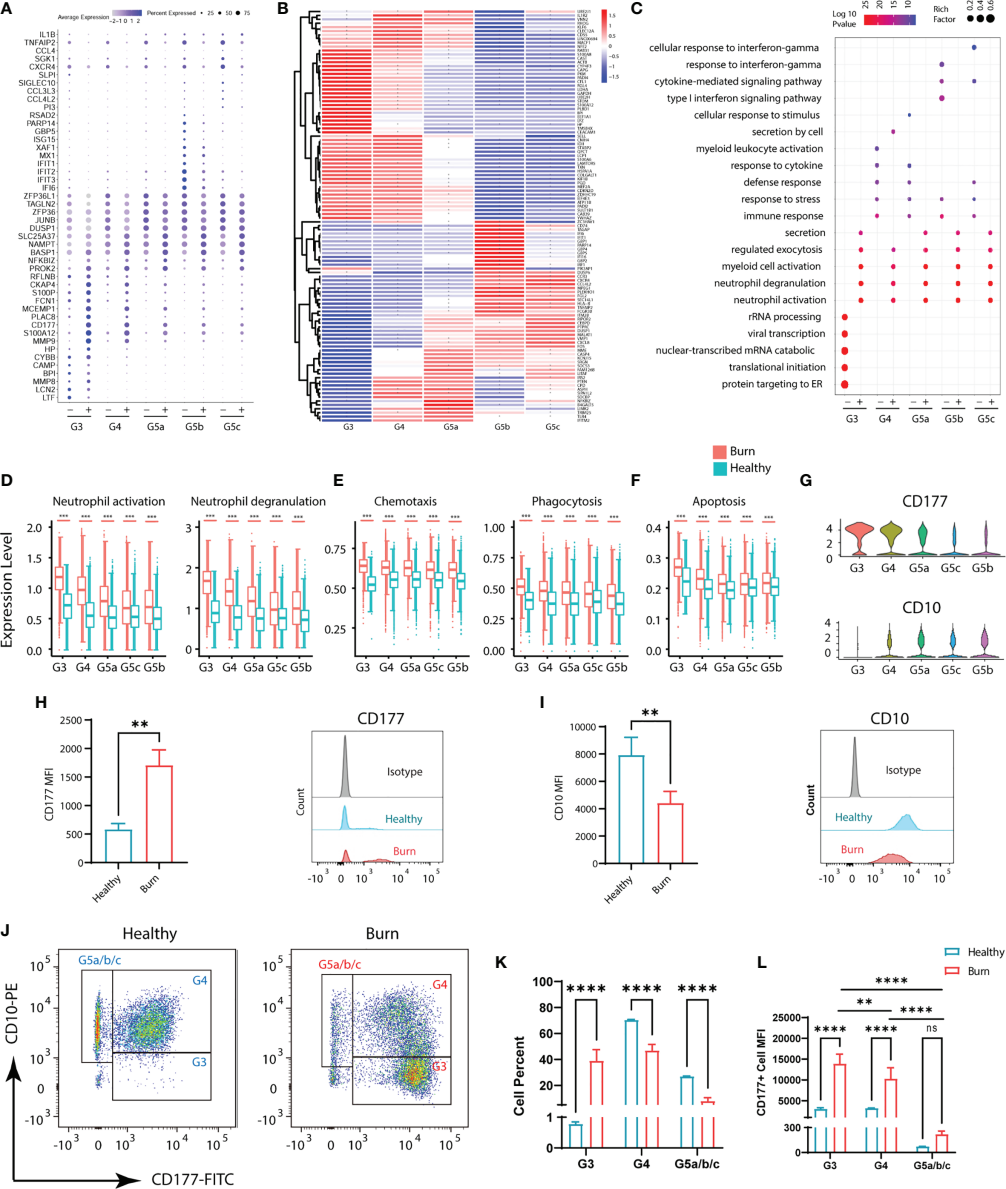
Functional changes of PMNs in healthy controls and severe burn injuries. **(A)** Conserved genetic tags of neutrophil subsets. **(B)** Heatmap of differential genes in subsets of PMNs in healthy controls and severely burn injuries. The asterisks represent differences in gene expression between burn injuries and healthy subjects. **(C)** GO (BP) analysis of neutrophil subsets in healthy controls or severe burn injuries. **(D–F)** Functional scores of each subgroup of PMNs. Neutrophil activation and neutrophil degranulation **(D)**, chemotaxis and phagocytosis **(E)**, apoptosis **(F)**. **(G)** Violin diagram of genes CD177 and CD10 in 5 subgroups. **(H–J)** The expression levels of CD177 and CD10 in circulating PMNs of healthy controls and early stages of severe burn injuries., Separation of G3, G4 and G5a/b/c PMNs by flow cytometry. FACS and staining strategy for G3 (CD10^low^-CD177^hi^), G4 (CD10^hi^-CD177^mid^) and G5a/b/c (CD10^hi^-CD177^low^) PMNs. **(K)** The proportion of each cell subgroup in the peripheral blood of healthy persons and burned patients. **(L)** Mean fluorescence intensity of CD177 expression on G3, G4,G5a/b/c subsets of PMNs in peripheral blood of healthy and burned patients. Data represent means ± s.d. (n= 3–8) of two independent experiments. **p < 0.01, ***p < 0.001, ****p < 0.0001, ns, not statistically.

The circulating PMNs in both burned patients and healthy controls was classified into the five cell subsets G3–4 and G5a–c, indicating the conserved nature of PMNs. Additionally, the identity of each neutrophil population was maintained during extensive burn injuries, and the signature genes still successfully indicated neutrophil identity (**Figure 3A**). However, the proportions of the five subgroups differed between the burned and healthy control groups. G3, G4 and G5a were the dominant subgroups in burned patients, whereas G5b and G5c were the dominant subgroups in healthy people (**Figure 2C**). The gene expression profiles and function significantly differed among the five clusters (**Figure 2B** and **Supplementary Figures 2A, B**). Clusters G5b and G5c were relatively senescent PMN subsets with high expression of aging markers (CXCR4). In addition, CCL family chemotactic ligands (CCL4, CCL3L3 and CCL4L2) were highly expressed among them. In contrast to the G5c subgroup, which showed high expression of enzyme inhibitor molecules (PI3 and SLPI), the G3 and G4 subgroups mainly showed expression of PMN activation markers (CD177) and many protease genes (**Figure 2A**).

To further predict the PMN function of each cluster, we performed GO analysis of the differentially expressed genes (DEGs), in categories including molecular function (MF), biological process (BP) and cellular component (CC). The BP terms indicated that the activation and degranulation functions of the G3 and G4 subgroups were significantly activated, as also confirmed by the related FunctionScore (**Figure 2D**). Degranulation of PMNs is accepted to be associated with the clustering of PMNs. Four types of granules exist in PMNs: azurophil or primary granules, specific or secondary granules, gelatinase granules and secretory vesicles (**Figure 2E**). We tested neutrophil granular contents in different PMN subgroups (**Figure 2F**). A significant increase was observed in cluster G3 in four levels of granules, particularly secondary granules (CYBA, CYBB and CAMP) and gelatinase granules (MMP8 and MMP9, **Figure 2G**). Beyond degranulation, each subgroup was rated for other functions, including neutrophil maturation, apoptosis, neutrophil aging, phagocytosis, necroptosis and chemotaxis (**Supplementary Figures 2C–H**). To study the directionality of each subgroup to disease, we scored disease function according to the DEGs (**Supplementary Table 1**). Cluster G3 was predictive of sepsis related diseases and inflammation, whereas infection was closely associated with the G5b subgroup (**Figure 2H**).

### Functional Changes in Subgroups in Severe Burn Injuries

Although the subgroups of PMNs were conserved, many genes underwent pronounced changes in severe burn injuries (**Figure 3B**). Through GO analysis of DEGs, we identified commonalities and idiosyncrasies across the five clusters. The results indicated that when the body was in a healthy state, these five subgroups were enriched in different GO entries, thus indicating that they perform different functions. In contrast, when the body experienced severe burn injuries, they were enriched in functions such as cellular activation, degranulation and exocrine secretion (**Figure 3C**). However, the degree of activation and degranulation of these groups was not identical (**Figure 3D** and **Supplementary Figure 3A–D**). G3 had the most significant degranulation and activation, followed by G4, and G5a–c also showed differences. Burn injury, a sudden and intense stimulus to the body, invokes a state of strong stress, which also leads to the stress response of PMNs, such as the up-regulation of chemotaxis and phagocytosis (**Figure 3E**). In parallel, genes associated with apoptosis were also up-regulated in response to strong flame burn injuries (**Figure 3F**).

CD177 is highly expressed in G3,G4 and serves as a marker of neutrophil activation (**Figure 3G**). By flow cytometry assay, we showed that CD177^hi^ PMNs and CD177^low^ PMNs were found in the peripheral blood of both normal and burned patients (**Figure 3H**). The difference is that there are more CD177^hi^ PMNs in the circulation of burned patients. CD10, a marker of granulocyte maturity, is significantly down-regulated in G3 subgroup (**Figures 3G, I**). In addition, we isolated PMNs based on their expression of CD10 and CD177. The group of G3 are relatively naive cells with low surface expression of CD10 and are overactivated PMNs with high expression of CD177. Thus, they were isolated as CD10^low^−CD177^hi^ PMNs by flow cytometry, while G4 cells were identified as CD10^hi^−CD177^mid^ PMNs. The remaining cells with high expression of CD10 and low expression of CD177 were considered to be relatively mature G5a/b/c subsets (**Figure 3J**). Through cell counting, we could find the rapid expansion of G3 subgroup in burned patients, which was also consistent with the results of single-cell sequencing (**Figure 3K**). At the same time, we also emphasized that the relatively naive PMNs have a higher degree of activation in burned patients (**Figure 3L**).

### Comprehensive Analysis of the G5b Subgroup

**Figure 4:**
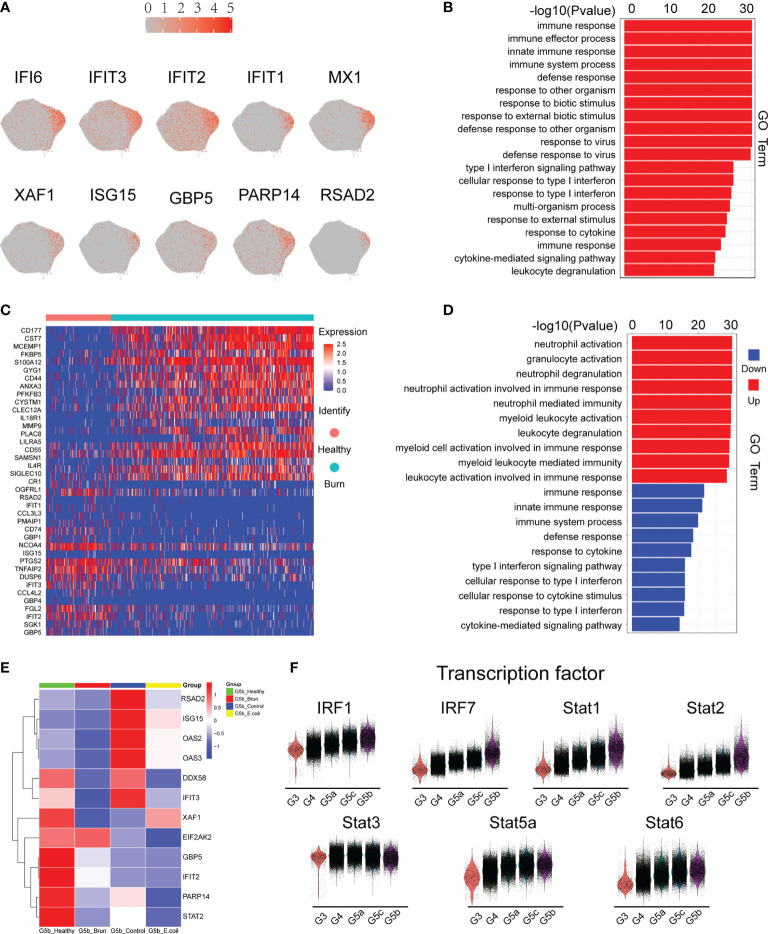
Characteristics of the G5b subgroup. **(A)** Specific genetic markers for the G5b subgroup. **(B)** Gene Ontology analysis of DEGs for the G5b subgroup compared with the other four subgroups. **(C)** Heatmap of differentially expressed genes in healthy and burned patients. **(D)** GO analysis (BP) of differential genes in healthy and burned patients. Red represents up-regulated differentially expressed gene cluster entries, and blue represents down-regulated differentially expressed gene cluster entries. **(E)** Heatmap of ISG gene expression in G5b of our study was compared with that of Xie et al. **(F)** Violin plot of characteristic transcription factors in the G5b subgroup.

G5b contains specific markers and expresses interferon-stimulated genes (ISGs; **Figure 4A**). Compared with other neutrophil subsets, G5b is involved in interferon-related pathways and plays a critical role in host defense (**Figure 4B**). Although G5b is a subgroup with type I interferon stimulation expression, activation and degranulation are its main functions in early stages of severe burn injuries (**Figures 4C, D**). After further subdivision according to the time of early burn injuries, we found that the function significantly differed between burn injuries on day 1 versus days 2 or 3 (**Supplementary Figures 4A, B**).

Previous studies have shown a rapid amplification of cluster G5b during E. coli infection (18). However, the amount of G5b decreased significantly in early stages of severe burn injuries (**Figure 6D**). The expression of ISGs also differed between infection and burn injuries (**Figure 4E**). The STAT transcription factor family regulates the transcription of various functional genes under the stimulation of different cytokines. STAT1 and STAT2, but not STAT3, STAT5a and STAT6, were genetic markers of G5b. Interferon specific regulators (IRF1 and IRF7) were also specifically overexpressed in G5b (**Figure 4F**).

### Abnormal Metabolism of PMNs in Early Stages of Severe Burn Injuries

**Figure 5:**
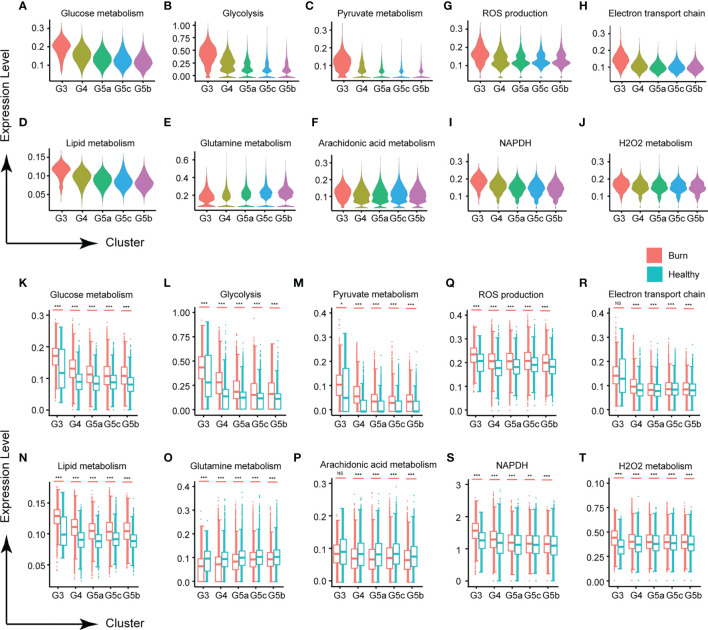
Changes in neutrophil metabolism in early stages of severe burn injuries. **(A–C)** Changes in glycometabolism-related functions in each subgroup. Glucose metabolism **(A)**, glycolysis **(B)** and pyruvate metabolism **(C)**. **(D–F)** Scores of lipid metabolism related functions of each subgroup. Lipid metabolism **(D)**, glutamine **(E)** and arachidonic acid metabolism **(F)**. **(G–I)** Changes in ROS metabolism-related functions in each subgroup. ROS production **(G)**, electron transport chain **(H)**, NADPH **(I)** and H_2_O_2_ metabolism **(J)**. **(K–T)** Changes in functions associated with glucose metabolism, lipid metabolism and ROS metabolism under healthy and burned conditions. *p < 0.05, **p < 0.01, ***p < 0.001, ns, not statistically.

Metabolic activity is a hallmark of living systems. Carbohydrate metabolism, lipid metabolism and ROS metabolism are the main metabolic types in PMN, which appear to be essential for the survival and activation of neutrophils. Under normal conditions, except for G3, the glucose metabolism of each subgroup of PMNs was relatively stable (**Figures 5A, K**). Although mitochondria are relatively absent in PMNs, and the main pathway of sugar metabolism is production of pyruvate through anaerobic glycolysis (**Figures 5B, C, L, M**), our results showed that PMNs are also involved in oxidative phosphorylation, the tricarboxylic acid cycle and the pentose phosphate pathway (**Supplementary Figures 5A–C**). Use of oxygen by neutrophils is widely recognized to be subject to a variety of physiological and pathological effects. When the body experiences a burn injury, each subgroup shows various manifestations of abnormal glucose metabolism, of which those in the G3 group are most serious.

Carbohydrate metabolism and lipid metabolism are inseparable in cell activities. In this study, PMNs also had abnormal lipid metabolism (**Figures 5D, N**). Glutamine, a conditionally essential amino acid, is associated with the biological functions of various immune cells (19). In early stages of severe burn injuries, the metabolism of glutamine and arachidonic acid in PMNs was significantly down-regulated, in contrast to the homeostasis of each subgroup’s metabolism observed under normal conditions (**Figures 5E, F, O–Q**). For other types of lipid metabolism, each subgroup showed no significant differences in homeostasis but showed significant activation after the burn injuries occurred, including phospholipid metabolism, neutral lipid metabolism, triglyceride metabolism, inositol phosphate metabolism and 3 phosphoadenosine 5 phosphosulfate metabolism (**Supplementary Figures 5D–H, I–P**).

Activated neutrophils are a major source of ROS, because PMNs contain multiple component complex enzymes (NAPDH oxidase) in the plasma membrane and phagosomal membrane. Burn injuries are usually accompanied by tissue and cell damage. Our findings demonstrated that burn injuries lead to ROS production and NAPDH oxidase activation (**Figures 5G, I, S** and **Supplementary Figures 5Q, S**). H_2_O_2_ and the electron transport chain, important components of the ROS, were also significantly up-regulated (**Figures 5H, J, R, T** and **Supplementary Figures 5R, T**).

### Dynamic Changes in Neutrophil Function in Early Stages of Severe Burn Injuries

**Figure 6:**
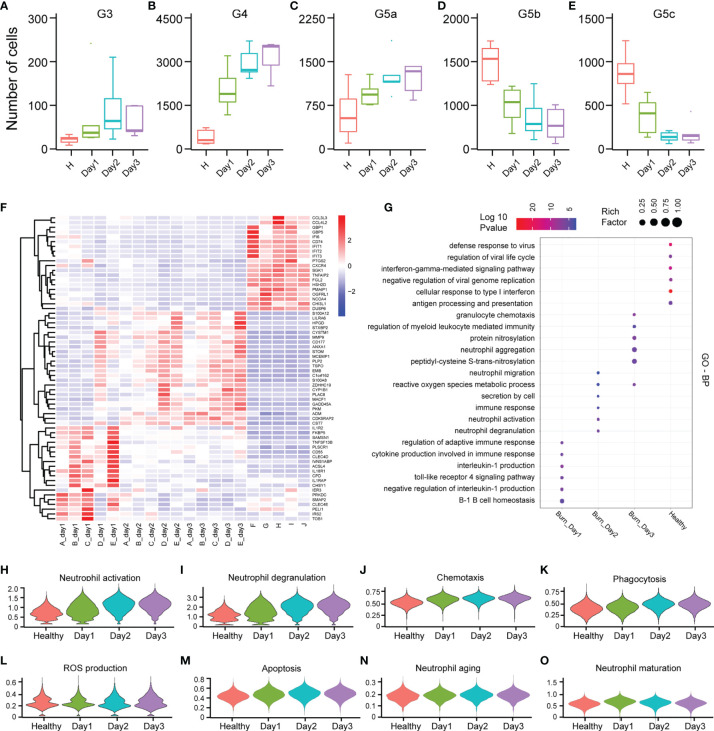
Differences in the functional characteristics of PMNs in the first 3 days of severe burn injuries. **(A–E)** Dynamic changes in the number of PMNs in each subgroup. **(F)** Heatmap of differential gene changes in 20 samples. **(G)** GO (BP) analysis results of PMNs in healthy controls and 3 days before severe burn injuries. **(H–O)** Violin plot of multiple functional changes in PMNs.

PMNs do not necessarily have constant function during early burn stages. To explore the functional changes in PMNs in early burn stages, we compared the functions of neutrophils on days 1, 2 and 3 after severe burn injuries. G3, G4 and G5a were the dominant subgroups of PMNs in burned patients. As the number of burned days increased, so did the number of G3, G4 and G5a (**Figures 6A–E**). Heatmap analysis indicated a significant difference between day 1 and day 2. However, in sharp contrast, day 2 and day 3 were not distinguished from each other (**Figure 6F**). To further predict the function of each subgroup, we performed GO analysis of the DEGs, including BP, MF and CC terms (**Figure 6G** and **Supplementary Figures 6A, B**). On the first day after burn injuries, cytokines and active immune regulation were the main factors involved, whereas on the second and third days, PMN activation, degranulation, chemotaxis, phagocytosis and ROS production were the main functions (**Figures 6H–L**). In other functions, including PMN metabolism, aging and maturation, no significant difference was observed across the 3 days (**Figures 6M–O** and **Supplementary Figures 6C, D**).

### Novel Transcription Factors in Severe Burn Injuries

**Figure 7:**
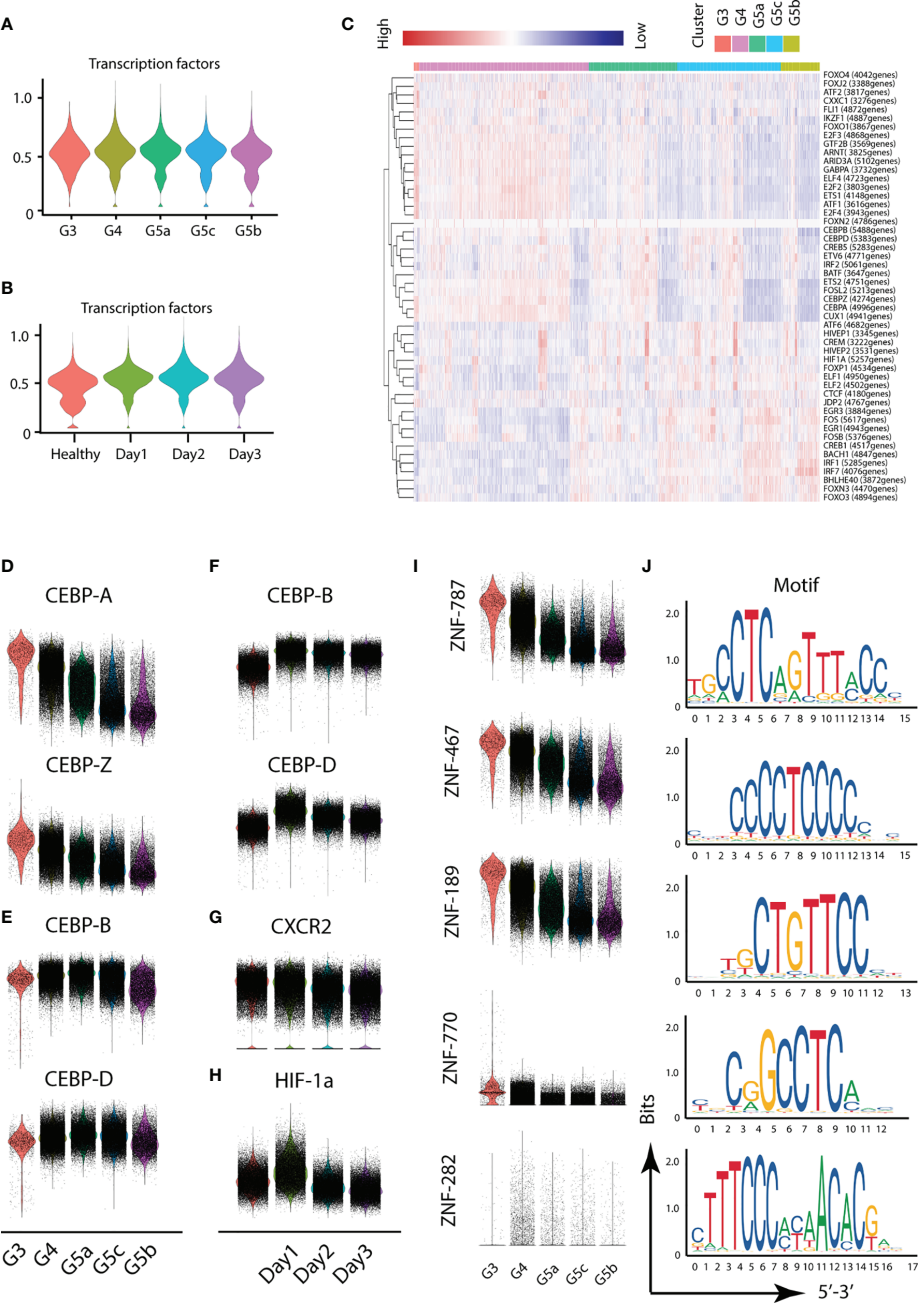
Transcriptional and communication characteristics of neutrophil subsets. **(A, B)** Violin plot of transcription factors. The transcription characteristics of PMNs in five subgroups **(A)**, and the transcription characteristics of PMNs in the healthy and burned groups **(B)**. **(C)** Heatmap of the difference in expression of transcription factors. **(D–H)** Differences in expression of related transcription factors under different conditions. **(I, J)** Expression of five novel transcription factors in each subgroup and prediction of the base sequence.

Different transcription factors are involved in the activation of different functions of PMNs. To probe the variation in transcription factors in severe burn injuries, we performed SCENIC analysis. Severe burn injuries induced an increase in the transcriptional activity of PMNs (**Figures 7A–C**). Members of the C/EBP family of transcription factors have been found to be necessary for neutrophil differentiation in neutrophil differentiation. The C/EBP-A transcript is highest in immature PMNs of the bone marrow, and C/EBP-B, C/EBP-D are abundant only in the most mature neutrophil precursors of the bone marrow and in peripheral blood granulocytes. Our scRNA-seq data indicated that the highest expression of C/EBP-A and C/EBP-Z transcription factors was in the G3 subgroup, whereas relatively high expression of C/EBP-B and C/EBP-D was observed in G4 and G5a–c (**Figures 7D, E**). This result indicated the consistency between our PMN grouping and traditional grouping. In addition, the expression of C/EBP-B and C/EBP-D was higher on day1 than days 2 and 3 (**Figure 7F**), in agreement with the role of bone marrow mobilization. Relatively mature PMNs were released from the bone marrow on the first day after severe burn injuries. Over time, relatively immature PMNs were mobilized. The expression of CXCR2, an indicator of bone marrow mobilization, was also confirmed (**Figure 7G**). Hypoxia-inducible transcription factors (HIF) participate in the body’s adaptive response to hypoxia. Overexpression of HIF-1a on day 1 implied the importance of adequate and prompt fluid resuscitation (**Figure 7H**). Unexpectedly, we also discovered five new transcription factors and that have been the subject of few studies, including ZNF-787, ZNF-467, ZNF-189, ZNF-770, ZNF-262. These transcription factors showed a clear expression difference across the five subgroups, thus indicating their importance (**Figures 7I, J**).

## Discussion

Severe burn injury is a complex pathophysiological process in which patients suddenly change from a relatively healthy state to a highly metabolic, highly exudative acute inflammatory state (20). Previous studies have described neutrophil dysfunction in the state of sepsis in the late stage of burn injuries, including delay in apoptosis, impairment of chemotactic function and overexpression of immunosuppressive molecules (21, 22). However, little attention has been paid to the functional changes in PMNs in early stages of severe burn injuries. Normally, PMNs constitute the first line of host defense against bacterial infections (23). PMNs are produced in the bone marrow and released to circulation within several hours after the burn injuries. The early mobilization of PMNs was not caused by the pathogens but was triggered by a strong stimulus such as heat damage (24). The roles of these circulating PMNs remain to be determined.

To further explore this question, we compared the population structures of peripheral blood cells by scRNA-seq in healthy people and those in early stages of severe burn injuries. Our analysis was based on the classification of PMNs in the study of Xie et al. (18). The difference between the studies was that, in addition to the relatively mature G5a, G5b and G5c subgroups, we also found G3 and G4 subgroups in the peripheral blood of healthy people. Among them, cluster G3 was an immature PMN subgroup, which showed stronger activation and degranulation functions than the other PMN subgroups. We found that the G3 subgroup was closely associated with sepsis and inflammation through disease prediction analysis. This result also coincided with those reported by Mare et al (21, 25). In addition, the G5b subgroup (a population with specific ISG gene markers) was predictive of infection status, and G5b had different functions in different disease states. G5b was dominated by the function of activation and degranulation under the strong stimulation of severe burn injuries. In the infected state, the G5b subgroup greatly expanded and played bactericidal and anti-infection roles. Our findings also suggested the conservation of PMNs. The heterogeneity of PMNs was not affected by severe burn injuries, and the expression of major identity markers was not significantly changed. However, the function of PMNs in the same subgroup may vary under different stimulation.

Commonalities and idiosyncrasies were found across the five clusters. The degree of degranulation and activation of each subpopulation increased in the burn state. G3 and G4, which are the dominant groups in early burn injuries, appeared to be more activated than other subgroups. Our previous studies have suggested that large amounts of particulate content, such as HBP and MPO, are produced by PMNs after burn injuries (8, 26, 27). HBP binds proteoglycans on the surfaces of endothelial cells, thus resulting in increased vascular leakage. In addition, MPO interacts with heparan sulfate glycosaminoglycan residues, thereby causing glycocalyx collapse and Sindcon-1 shedding. These results further demonstrate the unfavorable role of PMNs in early burn injuries.

In early burn stages, the organism is in a state of hypermetabolism, representing a potential hidden danger in the subsequent systemic inflammatory response. Immune cells undergo a metabolic switch depending on the extrinsic environment and disease state (28). Next, we performed in-depth studies on the metabolic function of PMNs. The corresponding abundance of metabolites and substrates (e.g., glucose, glutamine and oxygen) in the microenvironment appears to be critical for physiological survival and the activation of PMNs. Because of the lack of mitochondria, glucose metabolism becomes the main metabolic pathway in PMNs (29). An increase in glycolysis is expected in early burn stages because both cells and tissues are in a state of hypoxia. The main role of HIF-1a is promoting the metabolic adaptation to the hypoxic environment, as also confirmed by its upregulation on the first day after burn injuries. Additionally, previous studies have shown that glycolysis is positively correlated with PMN degranulation, in agreement with our results (30). Glutamine is a key substance enabling immune cells to perform normal functions, and the rate of consumption of glutamine is similar to or higher than that of glucose (19). Studies have shown excessive amino acid loss in the first 3 days after burn injuries, among which the highest loss is observed for glutamine (31). We observed that PMN glutamine metabolism was down-regulated in our burned patients, possibly because of insufficient organismal glutamine supply. The findings may be important to guide clinical supplementation of glutamine. In addition to glucose and lipid metabolism, ROS function was significantly up-regulated in burned patients, in terms of many aspects including NAPDH, H2O2 and the respiratory electron transport chain. In recent years, studies have shown that the production of trace amounts of ROS plays a regulatory role, particularly in signal transmission (32, 33). However, ROS have both negative and positive effects. Excessive ROS can damage a variety of biological components and even lead to DNA damage (34, 35). The destructive effect of ROS on vascular endothelial cells should be emphasized (36, 37). This mechanism is based on the strong oxidation of ROS, which damage several important functional enzymes in the plasma membrane in endothelial cells, including ion channels, and lead to dysregulation of water balance (36). Water molecules in blood vessels can enter tissues through endothelial cells and cause edema, consistently with the symptoms of high fluid exudation in patients with severe burn injuries. In the future, regulation of ROS release in PMNs is expected to become a new therapeutic target for alleviating the leakage of burned blood vessels.

To accurately control the functional changes in PMNs, we temporally subdivided the early burned period into days 1, 2 and 3. The transcriptomic landscape of PMNs in the 3 days varied in quality and quantity. On the one hand, the number of cluster G3, G4 and G5a increased with the passage of burned time, whereas the number of G5b and G5c gradually decreased. On the other hand, the functional changes in PMNs on the first day after burn injuries significantly differed from those at days 2 and 3. In terms of activation, degranulation and ROS, the changes in these functions were less dramatic on the first day after burn injuries than on the other two days, thus suggesting the possibility for clinical intervention. With advances in transportation and medical care, burned victims can generally be sent to the hospital for treatment on the first day. Simultaneously, early transition activation of PMNs potentially leads not only to a systemic inflammatory response but also to burn-related sepsis. Disease progression may be prevented by targeting PMNs on the first day after burn injuries. Unexpectedly, we identified transcription factors that have not been described in other studies and showed significant differences in expression across the five subpopulations of PMNs. There may be potential for further exploration of these transcription factors in the future.

In conclusion, ScRNA-seq was used to investigate circulating PMNs in early stages of severe burn injuries. PMNs were found to be relatively conserved, and PMNs in both healthy and burned patients could be divided into five subgroups with different genetic markers. However, each subgroup performed different functions in steady-state and under thermal stimuli. The dysfunction of circulating PMN in burned patients was mainly reflected in transition activation, degranulation and abnormal metabolic functions. Our findings may provide a valuable reference for subsequent treatment. In addition, the differentiation of PMN function in burned patients during the first 3 days also suggests the potential for clinical intervention to prevent subsequent serious complications.

## Materials and Methods

### Ethical Approval

This study was approved by The Medical Ethical Committee of Affiliated Suzhou Hospital of Nanjing Medical University. For experiments involving human blood samples, signed informed consent was obtained from all patients and healthy volunteers. Blood samples were taken from the cubital veins of patients and healthy donors. All the experimental methods were carried out in accordance with the approved guidelines.

### Human Sample Collection

Peripheral blood are taken from the healthy donors and burn patients. Blood samples from each healthy donor was collected into heparin anticoagulant tubes. Then, magnetic bead-based isolation kits were used according to the manufacturer’s instructions (Stemcell, Vancouver, Canada) to negatively select PMNs. The PMNs extracted by magnetic beads was washed once in 10ml of PBS (Gbico, Canada). Then centrifuge and remove the supernatant. PMNs were maintained in RPMI 1640 (Gbico, Canada) containing 10% fetal calf serum (FBS) (Gbico, New Zealand).

### Single-Cell RNA Sequencing

Single-cell transcriptome information was captured (from 20 sample sources) using the BD Rhapsody system. The single-cell suspension was randomly assigned to 200,000 micropores by a limited dilution method. The beads with oligonucleotide barcodes were added to the saturated state and paired with cells in the micropores. The cells were cleaved in micropores to hybridize mRNA molecules and the oligonucleotides on the beads were captured by bar code. Then reverse transcription and ExoI digestion were performed in a test tube. During cDNA synthesis, a unique molecular identifier (UMI) was bound to each cDNA molecule at the 5’ end to indicate the origin of the cell. The BD Rhapsody single-cell full transcriptome workflow includes random primer and extension (RPE), RPE amplification PCR and WTA index PCR to prepare a full transcriptome library. The high-sensitivity DNA chip (Agilent) on the bioanalyzer 2200 and the qubit high-sensitivity DNA analysis (Thermo Fisher Scientific) were used to quantify the library. Sequencing was performed by illumina sequencer (Illumina, San Diego, CA) on a 150 bp paired-end run.

### Single-Cell RNA Statistical Analysis

Fastp and default parameters are used to filter the adapter sequence and delete low-quality reads to obtain clean data. The single-cell transcriptome recognizes the white list of cell barcodes through the application UMI-tools (38). We map the data to the human genome (Ensemble version 91) by using STAR (39) mapping. The parameters were formulated by using the UMI-tools standard pipelinel to obtain the UMIs counts for each sample. We deleted the cell, which contained over 200 expressed genes and mitochondria UMI rate below 20% passed the cell quality filtering and mitochondria genes. Seurat software package (version: 3.1.4, https://satijalab.org/seurat/) was used to perform cell normalization and regression to obtain scaled data. The standard for PCA construction was the top 2000 high variable genes and the basis for the construction of tSNE and UMAP was the top 10 PCA. Since sample were processed and sequenced in batches, we used Harmony to remove potential batch effect.

We acquired the unsupervised cell cluster result based the top 10 principal through graph-based cluster method. The FindAllMarkers function and the Wilcox rank sum test algorithm were used to calculate the marker genes (lnFC> 0.25; pvalue <0.05; min.pct> 0.1).

### Flow Cytometry

Neutrophils were resuspended in precooled PBS to a density of 2×10^6 cells/mL (100 μL PBS, 2×10^5 cells/tube). CD177 and CD10 antibodies (BD Bioscience, USA) were used according to the manufacturer’s protocol. After incubating for 30 min at 4°C in the dark, cells were washed and detected in a FACS Canto II cytometer (BD Biosciences). The data were analyzed using the FlowJo software.

### SCENIC Analysis

The workflow of SCENIC based on three new R packages. First is GENIE3 which can identify potential TF targets based on co-expression. Second is RcisTarget which is to perform the TF-motif enrichment. Finally is AUCell, we use this R package to score the activity of regulons in single cells. We use the 20-thousand motifs database of RcisTarget and GRNboost to evaluate the regulatory strength of transcription factors and we also apply single-cell regulatory network and clustering workflow (pySCENIC, v0.9.5).

### QuSAGE Analysis (Gene Enrichment Analysis)

QuSAGE (40) (2.16.1) analysis we performed to represent the degree of activation of a given genome. QuSAGE is available as an R package now. We import Seurat Rdata to R and then use qusage function to analysis. After that using plotCIsGenes and plotGeneSetDistributions function to get the Visual results. We can find QuSAGE’s workflow in http://clip.med.yale.edu/qusage.

### Co-Regulated Gene Analysis

To discover the gene co-regulation network, find_gene_modules function of monocle3 (39) was used with the default parameters.

### Pseudo-Time Analysis

We applied the Single-Cell Trajectories analysis utilizing Monocle2 (http://cole-trapnell-lab.github.io/monocle-release) using DDR-Tree and default parameter. Before Monocle analysis, we select marker genes of the Seurat(version: 3.1.4) clustering result and raw expression counts of the cell passed filtering. On the basis of pseudo-temporal analysis, the branch expression analysis model (BEAM Analysis) was used to analyze branch fate-determining genes.

### Function Score

Scoring the gene expression of individual cells, these gene expressions represent certain biological functions. The average normalized expression of the corresponding gene is used to define the functional score.

### Identification of DEGs

We used the FindMarkers function (test.use=‘‘bimod’’, logfc.threshold=log[1.5], min.pct = 0.01) based on normalized data to identify DEGs. Gene Ontology analysis was performed by using the R package topGO. Disease Analysis was performed by a simlar-topGO method (Fisher’s Exact Test) based on DisGeNET (https://www.disgenet.org/).

## Data Availability Statement

The data presented in the study are deposited in the NCBI repository, accession number PRJNA772373. https://www.ncbi.nlm.nih.gov/bioproject/PRJNA772373.

## Ethics Statement

The studies involving human participants were reviewed and approved by The Medical Ethical Committee of Affiliated Suzhou Hospital of Nanjing Medical University. The patients/participants provided their written informed consent to participate in this study.

## Author Contributions

BS and JH designed the study and wrote paper. JH, ZZ, DJ, LLu, LLi, YC, and YS performed the RNA-seq data analysis. ZZ and DJ performed experiments. RS and YY performed collection of clinical data. JH and BS performed the statistical analysis. All authors contributed to the article and approved the submitted version.

## Funding

This study was supported by the National Natural Science Foundation of China, No. 82072217, 81772135 and U21A20370; by the Jiangsu Natural Science Foundation, No. BK20201178.

## Conflict of Interest

The authors declare that the research was conducted in the absence of any commercial or financial relationships that could be construed as a potential conflict of interest.

## Publisher’s Note

All claims expressed in this article are solely those of the authors and do not necessarily represent those of their affiliated organizations, or those of the publisher, the editors and the reviewers. Any product that may be evaluated in this article, or claim that may be made by its manufacturer, is not guaranteed or endorsed by the publisher.
