# Supplementary material for: Single-Cell Transcriptome Profiling Reveals Neutrophil Heterogeneity and Functional Multiplicity in the Early Stage of Severe Burn Patients
Source: Front Immunol. 2022 Jan 18;12:792122. doi: 10.3389/fimmu.2021.792122 (PMC8803731; doi:10.3389/fimmu.2021.792122)
Supplement: Supplementary file 1 [file DataSheet_1.docx]

**Supplementary Information for**

**Single-cell transcriptome profiling reveals neutrophil heterogeneity and functional multiplicity in the early stage of severe burn patients**

Jiamin Huang, Zhechen Zhu, Dongdong Ji, Ran Sun, Yunxi Yang, Lu Liu, Yiming Shao, Yi Chen, Linbin Li, Binwei Sun

*** Corresponding author**

Email: sunbinwei@hotmail.com

**This PDF file includes:**

Figures S1 to S6


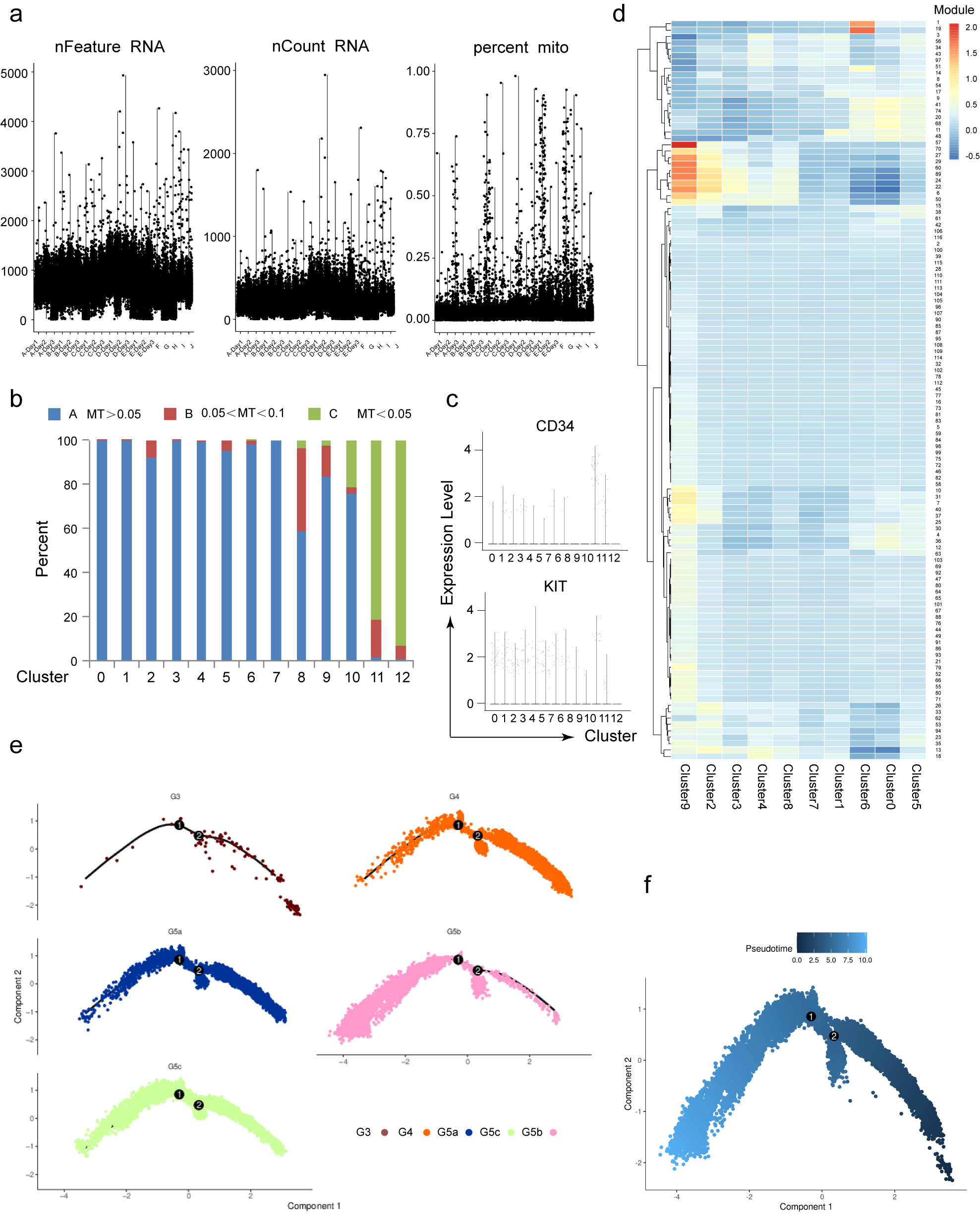


**Supplementary Fig. 1 Quality control and cluster correlation results related to SCLNA-SEq analysis.**

**a,** Violin diagram of the number of genes, number of UMI and percentage of mitochondria in 20 samples. **b**, The proportion information of mitochondrial genes in each subgroup, group A represents MT＞0.05, group B represents 0.05＜MT＜0.1, group C represents MT＜0.05. **c**, Violin diagram of genes CD34 and KIT in 13 subgroups. **d**, Gene Enrichment Analysis for each subgroup. e, Pseudo-time analysis results of cluster G3-G5C. **f**, Reference diagram of development direction for pseudo-time analysis. The positive direction of development ranges from dark blue to light blue.


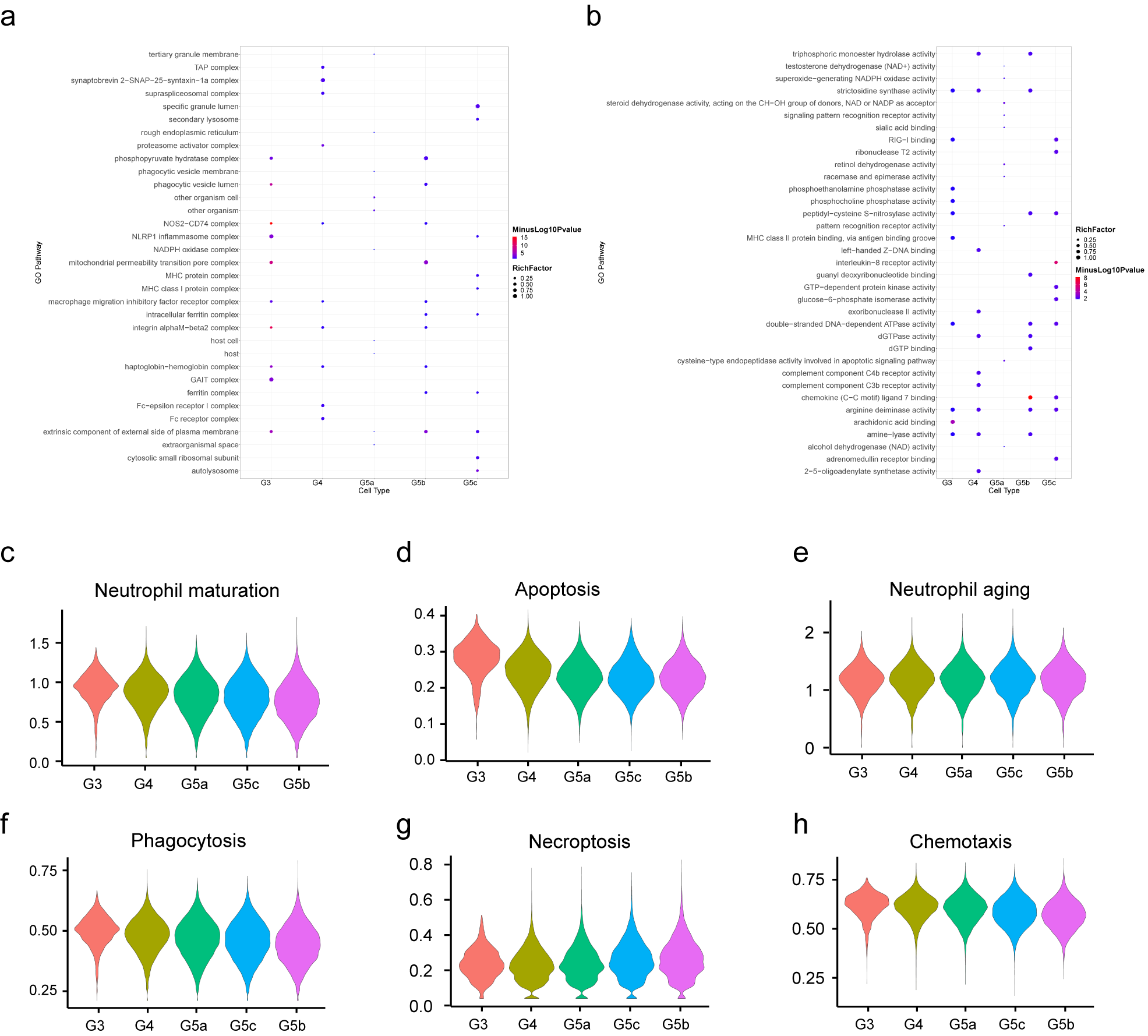


**Supplementary Fig. 2 GO analysis and related function scores of each subgroup of PMN.**

**a-b**, GO analysis of different genes in each subgroup, GO-CC analysis (left), GO-MF analysis (right) . The size of the circle represents the rich factor of genes, and the redder the color is, the higher the functional significance is. **c-h**, Violin chart of the scores of maturation, apoptosis, aging, phagocytosis, necroptosis and chemotaxis of neutrophils in 5 clusters.


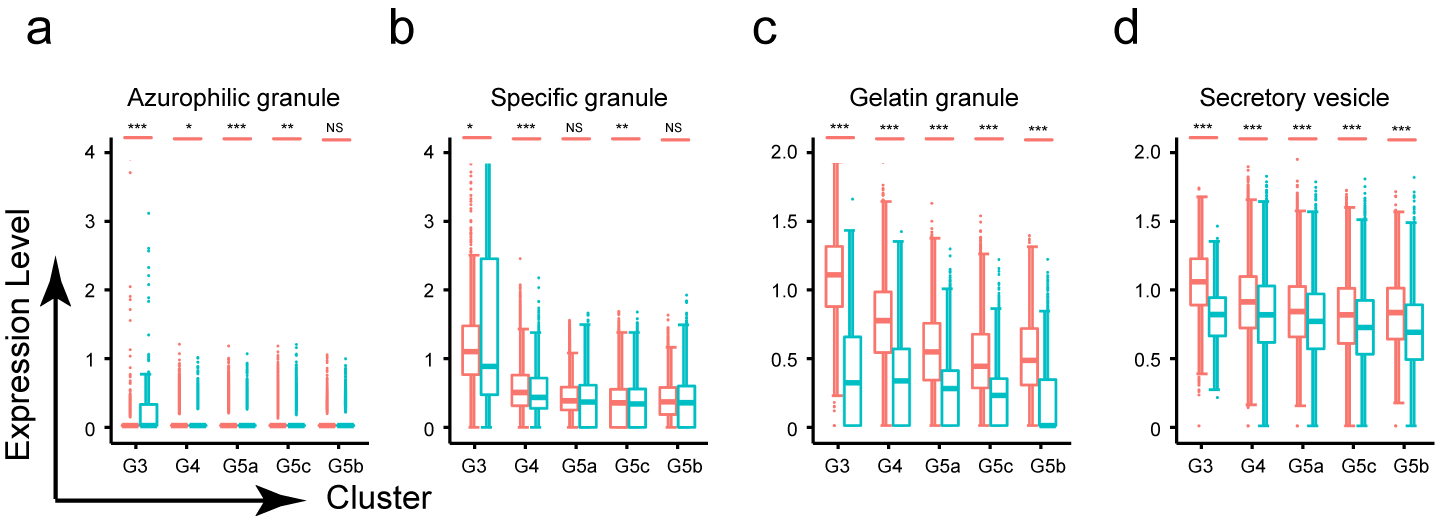


**Supplementary Fig. 3 Degranulation score of neutrophils in healthy controls and severe burns.**

**a-b**, Differences of four types of particulate matter in each subgroup in burn and health status. Azurophil granules (**a**), the specific granules (**b**), gelatinase granules (**c**), and secretory vesicles (**d**). *p < 0.05, **p < 0.01, ***p < 0.001, ns = not statistically, significantcompared with the healthy group.


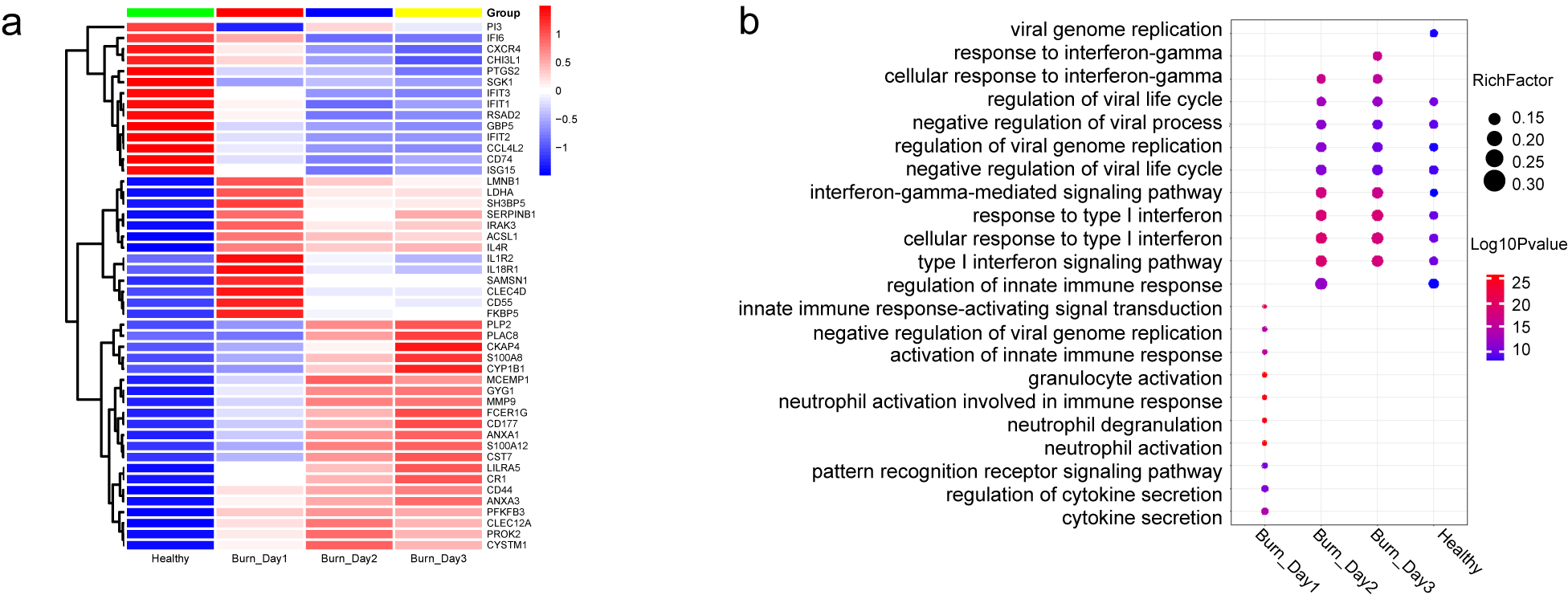


**Supplementary Fig. 4 Characterization of G5b subpopulation over time.**

a, Heatmap showing differential genes of subgroup G5b between healthy and burned patients (day1, day2, day3).

b, GO analysis (BP) of differential genes in healthy and burned patients (day1, day2, day3).


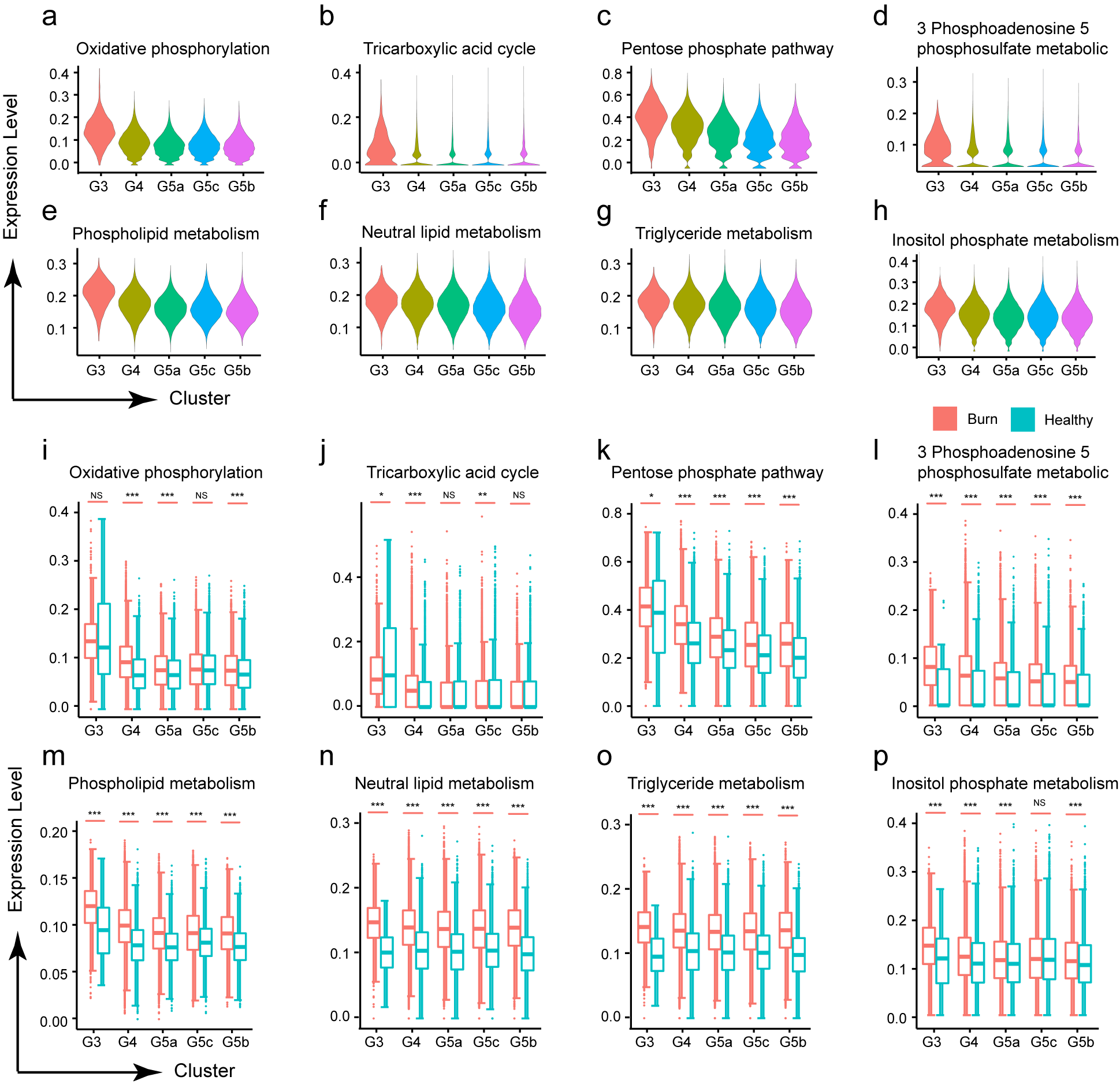


**Supplementary Fig. 5 Changes in glucose metabolism and lipid metabolism related functions.**

**a-h,** Scores of functions related to glucose metabolism and lipid metabolism in each subgroup. **i-p,** Changes in glucose metabolism and lipid metabolism between healthy and burned conditions.


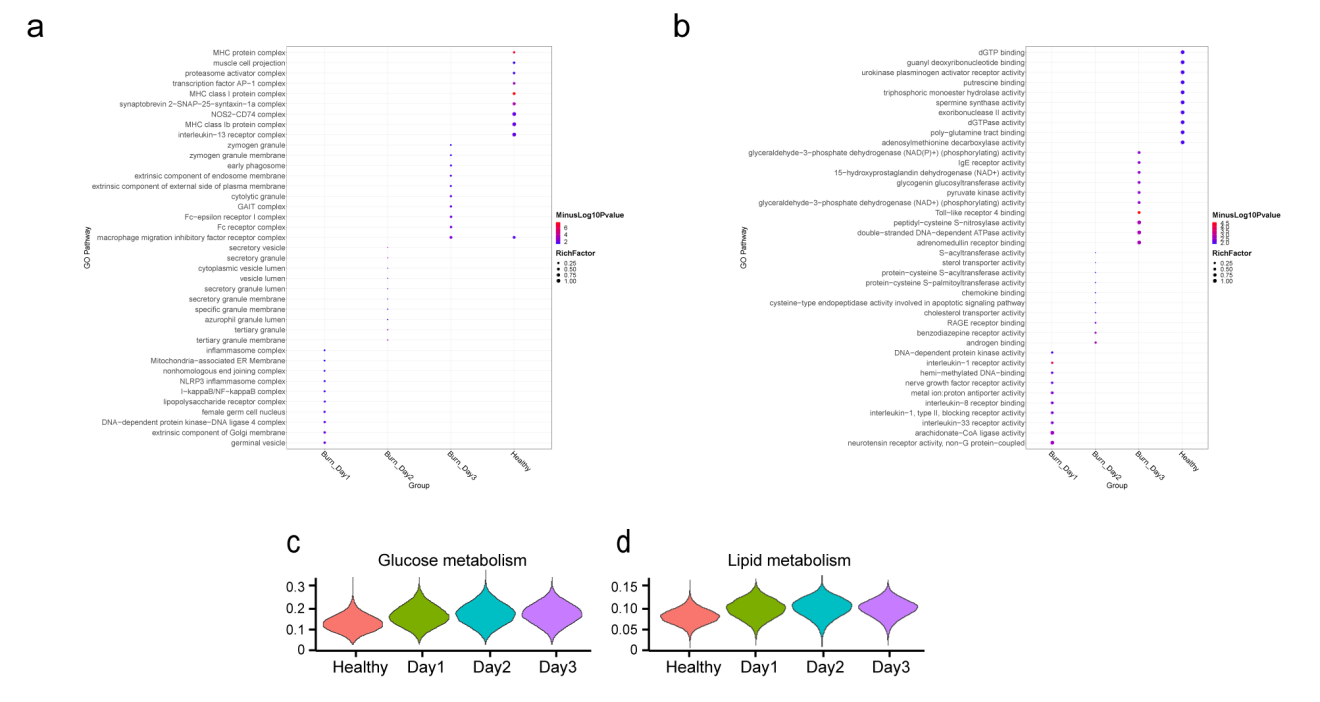


**Supplementary Fig. 6 GO analysis and functional score of PMN in the first three days of severe burn.**

**a-b**, GO analysis results of different groups. GO-CC analysis (left), GO-MF analysis (right) . **c-d**, Violin chart of functional scoring of glucose metabolism and lipid metabolism.
